# Building a Better Mouse Map

**DOI:** 10.1371/journal.pbio.0040422

**Published:** 2006-11-14

**Authors:** Mary Hoff

Genetic maps of the mouse genome—which identify the relative locations of specific stretches of DNA based on the likelihood of their being separated when chromosomes exchange parts during meiosis—work well for broadly defining where various points lie along a mouse’s chromosomes. But these maps have lacked the resolution that investigators need to be able to do things like line them up against physical maps—the string of As, Gs, Cs, and Ts that gene sequencing supplies—to identify the precise location of genes, or explore the nuances of genetic recombination. Because the mouse is a widely used model for genetic research, such capabilities would be invaluable. Now, Sagiv Shifman, Jonathan Flint, and colleagues have provided a powerful new tool for genetic studies with the development in mice of the most detailed genetic map available for any species but humans.

To create the high-resolution map, the researchers used two groups of mice, one consisting of outbred, heterogeneous stock (HS) and the other of recombinant inbred lines (RI). Physical mapping of the mouse genome has revealed the location of thousands of single nucleotide polymorphisms (SNPs)—stretches of DNA whose genetic code differs from one animal to another (or one homologous chromosome to another) by only one nucleotide base and that can be used as landmarks in the mapping process. The researchers looked at the patterns of inheritance of 10,202 SNPs in HS animals and 11,609 SNPs in RI animals, then used special software to calculate their relative location based on how likely they are to occur together. Using this process, they were able to create genetic maps of the mouse genome that can distinguish between two points 0.37 cM (centiMorgans, a measure of relative distance based on recombination frequency) apart in HS and 0.45 cM apart in RI—far more finely tuned than the best previous map.

After developing the super maps, the researchers used them to study recombination rates of various genes by comparing genetic and physical distances. For HS, the average recombination rate was 0.63 cM per megabase (cM/Mb), and for RI, it was 0.62 cM/Mb. But the recombination rate varied substantially from one part of the genome to another. Smaller chromosomes, for instance, had a higher average recombination rate than larger ones. There was also a difference between the study groups: the HS genome showed a higher recombination rate in big chromosomes and a lower rate in small chromosomes than did the RI genome. And when they looked at variation in recombination rate along the chromosome, the researchers found the highest recombination rate near the ends of the chromosomes (on structures called telomeres).

**Figure:**
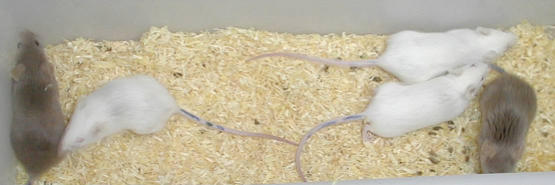
Genetically heterogeneous mice, derived from eight inbred strains, were used at the 50th generation for genetic mapping.

There was also a sex difference in recombination rates. Calculating rates separately for male and female HS mice, the researchers found, as previous studies had found in humans, that the average autosomal (non–sex chromosomes) recombination rate for females was higher than that for males. Distribution of recombination frequencies differed with sex, too, with recombination higher near the junctures of the sister chromosomes (centromeres) in females and higher near telomeres in males. The researchers also found many individual areas along the chromosomes that showed high recombination rates in one sex but not the other.

Intrigued by the incongruity in recombination, the researchers decided to look further into how rates vary with specific DNA features. In HS and RI together, they found a total of 494 regions in which recombination rates were uncharacteristically high (which they termed “jungles”) or low (“deserts”). The researchers looked at 55 inbred strains for places with little historical recombination. They found that 59% of deserts overlapped with such areas, while only 12% of jungles overlapped.

Can sequence characteristics predict jungles and deserts? In general, the researchers found more simple repeats but not more genes or SNPs in jungles. Self-copying stretches known as long interspersed nuclear elements (LINEs) were more common in deserts than in jungles. Sequences previously found to be prevalent in human recombination hot spots (CCTCCCT and CCCCACCCC) turned out to appear disproportionately often in the mouse genome jungles as well. In fact, the researchers found that the CCTCCCT motif appeared in locations corresponding to mouse jungles and deserts in rats, dogs, and chimpanzees, as well as in humans.

In the brief period of its existence, this new, improved mouse genetic map has already yielded valuable information on how factors such as chromosome, chromosomal location, sex, and sequence composition are related to recombination rates—information that can improve our understanding of inheritance and inform future efforts to pinpoint the precise location of genes on individual chromosomes.

